# Schistosomiasis outbreak investigation, Empandeni Ward, Mangwe District, Matabeleland South Province, June 2012; a case control study

**DOI:** 10.1186/1756-0500-7-623

**Published:** 2014-09-09

**Authors:** Pugie Tawanda Chimberengwa, Nyasha Masuka, Notion Tafara Gombe, Donewell Bangure, Mufuta Tshimanga, Lucia Takundwa

**Affiliations:** Department of Community Medicine, University of Zimbabwe, P.O. Box A178, Avondale, Harare, Zimbabwe; Provincial Medical Director, Matabeleland North Province, Mhlahlandlela Building, 10th Ave/Basch Street, PO Box 441, Bulawayo, Zimbabwe

**Keywords:** Schistosomiasis outbreak, Kwite village, Empandeni ward, Bloody urine, Zimbabwe

## Abstract

**Background:**

On 20^th^ of June 2012, 31 pupils from Kwite primary school reported to the local clinic complaining of passing bloody urine. A study was conducted to identify factors, the etiology and risks of contracting the disease.

**Methods:**

An unmatched 1:2 case control study was conducted at Kwite primary school. A case was defined as any child aged between seven to fifteen years, resident in Empandeni Ward for not less than two months, who had passed bloody urine with or without dysuria, fever, fatigue or lower abdominal pains from the 01/06/12 to 07/07/12. A control was a classmate of a case, staying in the same ward, who had not passed bloody urine. Controls were chosen by lottery method. A pretested questionnaire was administered to pupils and their caregivers. Environmental assessment was conducted; line lists, case notes, and district outbreak preparedness and response were reviewed using standard checklists.

**Results:**

All the 42 cases, and 84 controls were enrolled into the study. The median age for cases and controls was 10 years (Q_1_ = 9, Q_3_ = 12) and 10 years (Q_1_ = 8, Q_3_ = 11), respectively. Swimming in Kwite dam [AOR = 9.02, 95% CI (2.29-35.53)] and bathing in the dam [AOR = 3.22, 95% CI (1.10-9.41)] were independent factors associated with contracting schistosomiasis. *Schistosoma hematobium* was isolated in 31 out of 100 urine specimens examined. *Bulinus globosus* snails were identified at Kwite dam.

**Conclusion:**

The outbreak was driven by human contact with *S. hematobium* infested Kwite dam water, while poor knowledge on prevention of schistosomiasis by the Kwite community was evident. As a result of this study, health education to pupils and the community, mass drug administration on school pupils and mollusciding at the dam were done. The provincial health team adopted as on-going activities, the inclusion of schistosomiasis prevention and control in malaria pre-elimination activities.

## Background

Schistosomiasis, also known as bilharzia, is a chronic disease caused by parasitic trematode flatworms of the genus *Schistosoma spp.* [1, 2]. Worldwide over 230 million people require treatment for schistosomiasis yearly. Schistosomiasis is the second most prevalent parasitic disease worldwide following after malaria [1, 3]. Bilharzia is prevalent in poor communities without access to clean and safe water and poor sanitary facilities [2]. There are two types of schistosomiasis, intestinal and urogenital depending on presenting symptoms [2]. *S. hematobium* which causes urogenital bilharzia is more prevalent (20.8%) than *S. mansoni* (9%) in Zimbabwe [4].

When a person gets in contact with water contaminated with the parasite causing schistosomiasis, the parasite penetrates into the human skin and develops into adult worms in the human blood vessels [1, 5]. The worms mate and produce eggs which are passed in urine or stools [1]. The presenting signs and symptoms of schistosomiasis are caused by immunological reaction of the body against the worms as migrate through the body tissues and effects of eggs on epithelial mucosa [1, 3, 5, 6].

Intestinal schistosomiasis can result in abdominal pain, diarrhoea and blood in the stool while urinary schistosomiasis mainly presents with haematuria which may be associated with frequency and dysuria [2, 5]. Long term complications of schistosomiasis in adults include impotence, increased risk of bladder cancer, chronic kidney failure, kidney and bladder obstruction and heart failure [1–3, 5]. Children can develop anemia, stunting and reduced ability to learn [2]. Bilharzia is diagnosed through detection of parasitic eggs in stool or urine of cases [2, 5].

People at risk are those involved in agricultural, domestic and recreational activities [2]. Hygiene and play habit makes children vulnerable to the disease. Drinking clean water and adequate sanitation reduces the risk of contracting schistosomiasis [1, 2]. Control of schistosomiasis is based on drug treatment, snail control, improved sanitation and health education [1, 2]. Treatment should be complemented with access to safe and clean water, health education and good sanitation [1].

Mangwe district has been recording cases of schistosomiasis. This is shown by that the disease is ranked tenth among the top 10 out-patient commonly diagnosed diseases. Thus it features on the monthly line list at Kwite rural health centre. However, there were no outbreaks of schistosomiasis that have been reported in Mangwe district.

On the 20^th^ of June 2012, thirty-one pupils presented at Empandeni Rural Health Centre (RHC) complaining of passing blood in urine. The District Medical Officer for Mangwe was notified and a rapid response team (RRT) was sent for investigations on the same day. Thirty one urine specimens from all those that presented to the clinic were collected and sent to the Plumtree district hospital public health laboratory. Eighteen of the thirty-one samples tested positive for *S. hematobium* on microscopy.

This study was conducted to establish the factors associated with the schistosomiasis outbreak at Kwite Primary school. The belief of the community was that, there is no association between swimming and bathing in Kwite dam and contracting schistosomiasis. We hypothesized that swimming and bathing in the dam were associated with contracting schistosomiasis.

The study describes who was affected, when it occurred and how the outbreak occurred by linking the source of infection, the factors that were associated with contracting the disease and the level of knowledge of the pupils. We did determine the outbreak preparedness and response of the district health services while eliciting whether national treatment guidelines were adhered to in case management. The findings of the study are being used to assist in control and prevention of further schistosomiasis outbreaks in the Mangwe district and Matebeleland South Province at large.

## Methods

An unmatched 1:2 case-control study was conducted on pupils attending Kwite Primary School. A case was defined as any person aged between seven to fifteen years resident in Empandeni Ward for not less than two months, who has passed bloody urine with or without dysuria, fever, fatigue or lower abdominal pains between 1 June 2012 and the 7^th^ of July 2012. While a control, was any person aged between seven to fifteen years resident in Empandeni Ward for not less than two months, who has not passed bloody urine between the 1^st^ June 2012 and the 7^th^ of July 2012.

All children attending Kwite Primary school aged between 7 and 15 years who agreed to be interviewed and whose consent was sought from their legal guardians or parents and school authorities were enrolled into the study. All children who are below 7 years of age or in Grade one or below who were not able to respond to the questionnaire were excluded from the study. Children whose parents or they themselves disagreed to participate in the study were excluded.

The sample size was calculated using StatCal of Epi Info package. Assuming 30% exposure in controls and 50% exposure in cases that go for swimming in the dam, using Odds Ratio of 2.4 (Chirundu et al., 2005. unpublished), 95% confidence level, 80% power, and 10% refusal rate, a total of 212 cases comprising 106 cases and 106 controls were selected. However we recruited the whole population of 42 cases from the line list and powered up the study to two controls per case.

Sampling for controls was obtained by random sampling of pupils from the same class as cases using the lottery method. In this case, when (n) number of cases was chosen from a certain class, then (2n) number of controls were chosen from the same class using the lottery method. All pupils who were not cases in a class would draw papers marked ‘yes’ or ‘no’ from a hat. Only 2n papers were labeled ‘yes’ while the rest were marked ‘no’ so that all the pupils in each class with cases had an equal chance to participate in the study.

Data was collected using interviewer administered questionnaires for the study participants. A checklist was also administered at the rural health centre and the district for the district preparedness and outbreak response. An environmental assessment was carried out in the village, at the dam, other water sources and sanitary facilities.

Permission to carry out the study was sought from the Provincial Medical Director and Provincial Education Director for Matabeleland South; the District Medical Officer and the District Education Officer for Mangwe; Health Studies Office, school authorities and local village leadership. Ethical approval was obtained from the Joint Parirenyatwa Hospital and College of Health Sciences Research Ethics Committee and Medical Research Council of Zimbabwe. Written informed consent was obtained from the guardians or caregiver of the participants and their respective teachers. Confidentiality was maintained throughout and after the study. Names were not included on the questionnaire for confidentiality reasons.

## Results

There were 59.5% female cases and 40.5% male cases. There were 75% female controls. The cases and controls were not comparable by gender (p value <0, 01). The median age for both cases and controls was ten years, (Q_1_ = 9; Q_3_ = 12) and (Q_1_ = 8; Q_3_ = 11) respectively. The level of education was comparable for cases and controls (p = 0,533). Most cases were reported in grade 2 (31.8%) , median age 7 years, followed by grade 5 (23.8%) and the other grades had decreasing numbers of cases with the least being grade six with 4.8% of cases (median age 13 years). All the cases were from Kwite village. Controls and cases were comparable by village of origin (p = 0.602). Cases and controls were not comparable by religion (p <0, 01). On religion, 61.9% of the cases were from the apostolic sector while the majority of controls (42.9%) were from the Pentecostal church.

Table 1 below shows the factors associated with contracting bilharzias in Kwite village.

**Table 1:** **Factors associated with contracting bilharzia in Kwite village for cases and controls, June 2012**

Exposure		Cases n = 42 (%)	Controls n = 84 (%)	Odds ratio (95% CI)	p value
**Drinking water from an unprotected source**	Yes	39 (92.9)	64 (76.2)	4.06	0.022
	No	3 (7.1)	20 (23.8)	(1.13-14.57)	
**Swimming in dam**	Yes	39 (92.4)	32 (38.1)	21.13	<0.01
	No	3 (7.1)	52 (61.9)	(6.03-74.04)	
**Having garden at home**	Yes	27 (64.3)	64 (76.2)	0.57	0.159
	No	15 (35.7)	20 (23.8)	(0.25-1.26)	
**Bathing in the dam**	Yes	31 (73.8)	21 (25)	8.45	<0.01
	No	11 (26.2)	63 (75)	(3.63-19.72)	
**Using toilet at home**	Yes	21 (50)	50 (59,5)	0.68	0.310
	No	21 (50)	34 (40.5)	(0.32-1.43)	
**Fishing in dam**	Yes	28 (66.7)	22 (26.2)	5.63	<0.01
	No	14 (13.3)	62 (73.8)	(2.59-12.61)	
**Crossing water body to school**	Yes	32 (76.2)	45 (53.6)	2.77	0.014
	No	10 (23.8)	39 (46.4)	(1.21-6.36)	
**passing human waste into water bodies**	Yes	20 (47.6)	19 (22.9)	3.06	0.005
	No	22 (52.4)	64 (77.1)	(1.39-6.77)	

The majority of cases (92.9%) and controls (76.2%) did drink water from an unprotected source, and this was significantly associated with contracting schistosomiasis [OR = 4.06 (CI = 1.13; 14.57)]. Those that took a bath in the dam were 8.45 times more likely to develop schistosomiasis than those who did not bath in the dam. Pupils that went swimming in the dam were 21 times more likely to develop the disease than those who did not. Fishing in the dam, crossing through water bodies to and from school and passing human waste into the open water bodies were significant risk factors for contracting schistosomiasis. However using a toilet was protective of contracting schistosomiasis [OR = 0.68(0.32; 1.43)] although it was not statistically significant (p = 0,31).

The independent factors associated with contracting bilharzias are shown in Table 2.

**Table 2:** **Logistic regression for factors independently associated with contracting schistosomiasis in Kwite, June 2012**

Factor	Adjusted OR (95% CI)	p-value
**Swimming in dam**	9.017 (2.29-35.53)	0.0017
**Bathing in dam**	3.221 (1.10-9.41)	0.0326

Swimming in the dam [AOR = 9.017 (CI = 2.29; 35.53)] and bathing in the dam [AOR = 3.22 (CI = 1.1; 9.41)] were factors that were independently associated with contracting schistosomiasis in Kwite village. Of the cases, 44% did swim for more than 4 times a week preceding schistosomiasis infection.

**Figure 1:**
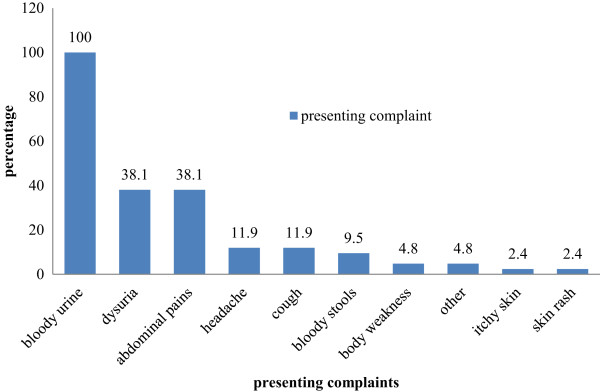
**Presenting signs and symptoms for cases with Schistosomiasis, Kwite, June 2012.**

Figure 1 below shows the presenting signs and symptoms for cases that presented with schistosomiasis in Kwite village.

All the cases reported passing blood in urine. Thirty two percent of the cases did complain of abdominal pains and dysuria. There were 11.9% who reported experiencing headaches and cough. Other associated symptoms were not very common body weakness (4, 8%), itchy skin and skin rash (2, 4%). There were however 9.5% of cases who reported passing bloody stools.

On the 20^th^ of June 2012, thirty one urine samples were collected from pupils who had visited the clinic. 18 samples were positive for *S. hematobium* on microscopy. On the 22^nd^ of June 2012, sixty-nine more urine samples were collected at the school and 11 samples were positive. Thus this is the total of 42 cases that were enrolled into the study.

All the cases were treated according to standard treatment guidelines with praziquantel 40 mg/kg body weight [7]. There was no symptomatic management which was done although the medicines such as antibiotics and analgesics were in the clinic. Only antihistamines were not available at the rural health centre though they were available in the district pharmacy.

Knowledge of pupils on the causes and signs and symptoms of the disease was satisfactory. About 95% of cases and 90% of controls managed to link the cause of schistosomiasis to infested water source or snails with only 4, 8% of cases not knowing what causes schistosomiasis. Only 16.7% of cases knew that avoiding infested water contact would help in preventing contracting schistosomiasis. Zero percent of cases and 1,2% of controls knew that washing hands after toilet use does help to prevent spread of disease.

An environmental assessment was carried out at the dam as it was a potential source of the infection. The dam is 300 meters away from the school grounds. At the dam, scooping for snails using a flat dip net scoop was done. Snails of *Bulinus globosus* species were collected and identified in the public health laboratory at Plumtree District Hospital.

## Discussion

There was a general increase in trend of cases from 7-12 years then decline among older ages. Gryseels et al. (2006) and Ndamba et al. (1998) reported that those aged between 8-15 years most affected with schistosomiasis with higher infection rates in children than in adults [8, 9]. In a study done in Kenya, Shimada et al. (1987), reported the highest prevalence of schistosomiasis is among children and young adults in many communities, because they are more likely to frequently use river sources for recreational purposes [10]. In our study, there were more females than there were males. This was possibly because the female pupils spent more time in contact with contaminated dam water while swimming, washing, bathing and they also did fishing using nets [11, 12].

There were significant associations between having a current schistosomiasis infection and participating in swimming, bathing in the dam, fishing using a fishing line and fishing with legs in water. This shares similar findings with a water contact observation study done by Chandiwana et al. (1987) where water contact activities such as bathing and recreational and personal use were identified as the risks of contracting schistosomiasis [13]. Other activities in Kwite such as brick molding and watering the garden using dam water were all common practices. We also noted that the school pupils did go for swimming in the dam during break time and after school, they did fetch water for the school garden from the dam. These activities predisposed the school children to getting infected with schistosomiasis.

We observed *S. hematobium* in urine 31% of samples that were collected. Of note is that 9.5% of cases alluded to having passed bloody stools and the isolation of *Biomphalaria pfeifferi* snail species in Kwite dam was suggestive that *S. mansoni* could be a possible cause of schistosomiasis in Kwite. Stool samples could have been done to confirm S.mansoni in Kwite. *Bulinus globosus* snails that were seen were responsible for transmission of *S. hematobium*. Midzi et al. (2014), reported that S*. hematobium* was more widely distributed in Matabeleland South province [14].

Knowledge levels on causes of disease were high among the pupils while it was lacking on prevention of schistosomiasis. There is thus need to spread messages through health education aided by IEC materials in Kwite. Educational programs can improve knowledge about the disease and healthcare seeking, but behavior can be difficult to change without other options for water contact [8]. The Kwite village’s main source of water for drinking was from the dam during the preceding six months as the three boreholes in the area had broken down. The toilet coverage in the village was lowest at 49% compared to an average of 80% in other villages of Empandeni ward. The provision of safe water supplies and latrines is useful, but for the prevention of schistosomiasis, safe contact sites are also needed [8].

To control schistosomiasis, strategies include indiscriminate mass treatment, snail control, active case finding, and treatment of particular risk groups such as school-aged children, snail control and health education [1, 2, 15]. However, there were no Information Education and Counseling materials specifically for schistosomiasis the health centre or in the community.

Population-based treatment is feasible, safe, and effective [8]. The main technical difficulty lies in identification of remaining cases and pockets through an integrated surveillance and response system [13]. Mollusciding was done in Kwite dam though surveillance need to be maintained. Molluscides used to eliminate snails in freshwater sources may harm other aquatic lives and, if treatment is not sustained, the snails may return to those sites afterwards [1]. Therefore there is need to maintain surveillance on snail populations on the same note identifying biological methods of controlling snails [4].

The district was well prepared for the outbreak as surveillance meetings were being held at the rural health centre and at the district. Surveillance minutes and schistosomiasis line lists at the rural health centre and the district offices were seen and verified to be up to date. Integrated disease surveillance and response trainings were done to the district managers but were not extended to the rural health centre staff.

The outbreak response by the district was as per stipulated guidelines. The district was notified by the health centre per phone on the 20^th^ of June 2012. The DMO activated the rapid response team on the same day which was dispatched to investigate the suspected outbreak. Within 48 hours a concrete response was mounted by the district outbreak investigation team at Kwite village.

All 242 pupils present on the day were given Mass Drug Administration (MDA) with praziquantel. Mollusciding was done at the dam to control snails. Health education was intensified to both school pupils and the community on the need to avoid human-contaminated water interface [16, 17]. Follow up of schistosomiasis surveillance was taken up by the district health officials who were scheduled to conduct repeat MDA and controlling snails in the Kwite dam. The provincial health team adopted as on-going activities, the inclusion of schistosomiasis prevention and control in malaria pre-elimination activities.

## Conclusion

In conclusion, the schistosomiasis outbreak among pupils attending Kwite primary school affected more female pupils than male pupils. The outbreak was caused by *S. hematobium*. Kwite dam was the source of infection where human-contaminated water interface through swimming and bathing in the dam was the primary mode of transmission. Bilharzia was managed according to national treatment guidelines using praziquantel.
